# Eligibility for Human Leukocyte Antigen–Based Therapeutics by Race and Ethnicity

**DOI:** 10.1001/jamanetworkopen.2023.38612

**Published:** 2023-10-26

**Authors:** Timothée Olivier, Alyson Haslam, Jordan Tuia, Vinay Prasad

**Affiliations:** 1 Department of Oncology, Geneva University Hospital, Geneva, Switzerland; 2 Department of Epidemiology and Biostatistics, University of California, San Francisco

## Abstract

**Importance:**

The development of therapeutics for patients who are positive for specific human leukocyte antigen (HLA) subtypes evokes the question of whether certain racial and ethnic groups are more or less likely to be eligible for novel products.

**Objective:**

To determine whether racial and ethnic inequities were present with regard to trial eligibility in trials investigating a therapeutic restricted to patients with specific HLA subtypes.

**Design, Setting, and Participants:**

This cross-sectional study included all clinical trials registered in ClinicalTrials.gov through March 18, 2022, that investigated an interventional study of a therapeutic strategy and restricted participants to those with at least 1 HLA subtype. Data were analyzed from May 8 to July 1, 2022.

**Main Outcomes and Measures:**

The type of therapeutics used in trials, the condition under study, the HLA subtypes used, and the likelihood of being enrolled in such a trial according to race and ethnicity.

**Results:**

Of 2135 trials identified, 263 met inclusion criteria. Overall, the estimated likelihood of being eligible for an HLA-based trial was 50.3%. Individuals of African American descent had the lowest likelihood of eligibility (33.0%), while being an individual of European descent conferred the highest (53.0%; 1.6 times more likely than African American individuals). Most trials studied anticancer therapeutics (258 [98.1%; 95% CI, 96.4%-99.7%]), and most were a therapeutic vaccine (179 [68.1%; 95% CI, 62.4%-73.7%]). The HLA-A*02:01 allele and the HLA-A2 serotype were the most frequent HLA subtypes for trial eligibility. The frequency of the HLA-A*02:01 allele in the population varied, with 11.9% (95% CI, 11.8%-12.0%) in African or African American individuals and 27.1% (95% CI, 27.1%-27.1%) in individuals of European descent.

**Conclusions and Relevance:**

The findings of this cross-sectional study suggest that enrollment restrictions for clinical trials investigating novel HLA therapeutics may be associated with racial and ethnic inequities with regard to trial eligibility. Overcoming these restrictions poses biological challenges, but solutions must be implemented to provide equal access to innovative strategies regardless of race or ethnicity.

## Key Points

**Question:**

Is availability of therapeutics for patients with specific human leukocyte antigen (HLA) subtypes associated with inequalities by race and ethnicity?

**Findings:**

In this cross-sectional study that included 263 trials investigating HLA-restricted therapeutics, the likelihood of being eligible for such trials was unequal according to racial or ethnic background, with African American individuals having the lowest likelihood of eligibility and individuals of European descent having the highest likelihood of eligibility.

**Meaning:**

These findings suggest that overcoming HLA restrictions in clinical trials poses biological challenges, but solutions must be implemented to provide equal access to innovative strategies regardless of race and ethnicity.

## Teaser

This cross-sectional study assesses enrollment restrictions for clinical trials investigating novel therapeutics for certain human leukocyte antigen (HLA) subtypes and compares these findings with the global prevalence of the most common HLA subtypes by racial and ethnic group.

## Introduction

In recent years, several drugs underwent development only for individuals who have specific human leukocyte antigen (HLA) subtypes. These HLA subtypes are a protective mechanism that ensures the integrity of individuals (the self) against external threats (the nonself).^1,2,3^ The HLA complex is composed of hundreds of highly polymorphic genes located on the short arm (p) of the sixth chromosome.^2,3,4^ Given the unique role these molecules play in the immune system, some drugs have been engineered to bind to specific HLA constructs, and many more are in the pipeline. One drug, tebentafusp, a fusion protein targeting gp100, has received US Food and Drug Administration approval in advanced or metastatic uveal melanoma, only for patients positive for HLA-A*02:01.^5,6^

Frequencies of HLA alleles vary by race and ethnicity.^4^ Thus, the development of therapeutic products being restricted to individuals bearing specific HLA subtypes raises the question of whether structural biases may be occurring—that is, whether some ethnic or racial groups are more or less likely to be eligible for novel products.

We sought to systematically describe characteristics of clinical trials investigating therapy targeting patients with specific HLA subtypes. We identified which HLA subtypes were used in each trial and compared our findings with the global prevalence of the most common HLA subtypes by racial and ethnic groups.

## Methods

### Study Design and Research Strategy

In this cross-sectional study, we sought to identify all clinical trials investigating a therapeutic strategy (drugs, biologics, cellular therapy, vaccine, and other) and restricting participant enrollment to 1 or several HLA subtypes. We then compared these types against population HLA frequency. We adhered to the Strengthening the Reporting of Observational Studies in Epidemiology (STROBE) reporting guideline. Because we used publicly available data, and because the study is not considered human participant research in accordance with 45 CFR §46.102(f), we did not submit this study to an institutional review board, nor did we require informed consent.

### Definition and Nomenclature for HLA Serotype and Subtypes (or Alleles)

The research was conducted through the ClinicalTrials.gov search engine using the keyword *HLA* in the “other terms” category. Historically, HLA molecules were mainly identified through serotype testing and cellular assays but were gradually replaced by molecular analysis.^7^ Serologic assays discriminate a limited number of epitopes on the surface of HLA molecules defining serotypes (eg, HLA-A2), compared with molecular biology, which targets up to a single nucleotide difference, thus enabling identification of alleles with 4 levels of definition (eg, HLA-A*02:01:01:03).^7^ Due to progressive incorporation of new understanding and technical advances over time, the matching between serotypes and alleles is not always straightforward, as illustrated by dictionaries listing HLA serotypes with their corresponding alleles.^8^ Important and continuous efforts have been made to mitigate challenges posed by HLA allele classification worldwide.^9^

In our analysis, trials could refer to either serotypes or alleles in their inclusion criteria, and we denoted the term *subtype* to encompass both entities. For instance, some trials were studying a drug for patients positive for HLA-A2 (a serotype), and others in patients positive for HLA*A-02:01 (an allele that is included in HLA-A2 serotype family). Both are termed *subtypes* in our work.

### Inclusion and Exclusion Criteria

Trials needed to (1) investigate a therapeutic strategy, (2) be an interventional study (clinical trial) or expanded-access study, and (3) restrict participants to an HLA subtype in at least 1 study group. Studies could be of any status of enrollment (active, not yet active, terminated, or other).

We excluded (1) preclinical data, (2) nontherapeutic intervention trials and observational studies, (3) trials focusing on drug sensitivity related to HLA subtypes, (4) trials using HLA subtypes in the inclusion criteria to select for an HLA-related disease (eg, celiac disease or type 1 diabetes), and (5) studies in which the specific HLA subtypes were not stipulated (eg, transplant trials or HLA-matched strategies). No restriction was placed regarding inclusion period.

Searches were performed on March 18, 2022. The study selection was performed independently by 2 reviewers (T.O. and A.H.).

### Data Abstraction

For each identified trial, we abstracted the NCT identifier number, the phase of the trial, the disease or condition treated, including the tumor type if the condition was cancer, the starting date of the trial, the enrollment status (active, not yet active, terminated, or other), the number of patients expected to be enrolled, the HLA-serotypes or subtypes used, whether there was 1 or more than 1 HLA subtype, and the mechanism of action of the intervention. To classify the intervention according to the mechanism of action, we used broad categories:

1. *Therapeutic vaccines* comprised any kind of therapeutic vaccine (including epitope-based, dendritic cells–based, DNA vaccine, or others).^10^
2. *Cellular therapy* was used for every strategy using cell engineering, including CAR-T cells.^11,12^
3. *Fusion protein* was defined as, for example, tebentafusp.^5,6^

### Prevalence of HLA Subtype According to Racial and Ethnic Groups

For HLA subtypes identified in trials, we sought to estimate the prevalence of those among 7 racial and ethnic categories (African or African American; American Indian or Alaska Native; Asian or Pacific Islander; European or European descent; Middle Eastern or Northern Africa; South or Central American, Hispanic, or Latino; and unknown, not asked, multiple ancestries, or other). We used the catalog of Common, Intermediate and Well-Documented (CIWD) HLA Alleles in World Populations, version 3.0.0. This is one of the most comprehensive and updated databases of HLA alleles worldwide, with data derived from more than 8 million individuals classified into 7 geographic, ancestral, and racial and ethnic origins.^4^ This data set combines multiple registries, and races and ethnicities were self-reported and encoded by each individual registry. The data were published in 2020 and raw data were extracted from the 18th International HLA & Immunogenetics Workshop website.^13^

In this study, allele frequencies were used to estimate prevalences within populations. The prevalence can range from as low as the allele frequency, if all individuals are homozygous, to as high as twice the allele frequency, if all individuals are heterozygous. Because of the high polymorphism of the HLA system, most individuals are heterozygous, and we estimated the prevalence as twice the allele frequency. This is the highest projection and has the potential for overestimation. However, our estimates aligned with direct prevalence data. We used this method because direct prevalence data according to race or ethnicity were unavailable in the CIWD data.

Race and ethnicity are different in definition; however, they are often closely intertwined due to historical and social factors. One’s race and ethnicity can overlap, but they can also be distinct. For example, someone might be racially categorized as Black and ethnically identify as Jamaican or Ethiopian. We used both terms in this study because some abstracted data were based on ethnicity (eg, the CIWD) while others were based on race (ClinicalTrials.gov). Whenever possible, we closely matched, the terms with the database from which the data were provided.^14^

### Likelihood of Being Enrolled in Trials

Based on the identified HLA subtypes in each included study and the number of slots for each trial, we calculated the total number of slots for all included trials and the proportion of slots available for each identified HLA subtype. Following the principles of HLA classification, the numbers of slots available for HLA serotypes were calculated, if necessary, by adding slots available for the serotype (eg, HLA-A2) to slots available for patients with alleles included in the serotype (eg, HLA*A-02:01 or HLA*A-02:06).

Based on the calculated proportion of slots available for each HLA subtype and the estimated prevalence of this subtype across racial and ethnic groups, we calculated the likelihood (percentage) of being enrolled in a trial for each racial and ethnic population and the overall population. As an example of calculation, the HLA-A2 serotype was a criterion for inclusion in 84.9% of all the trials. Among African American individuals, this serotype allele frequency is 18.6%, thus the prevalence was estimated to be 37.2% of individuals (maximum estimate). This resulted in a 31.6% probability that an African American individual would be eligible for trials restricting to those with the HLA-A2 serotype. We applied similar calculations for all the HLA subtypes we identified in our study. For example, HLA-A24 was a selection criterion in 4.0% of trials. Given a 2.9% allele frequency of this subtype in African American individuals, there's a 0.2% likelihood they would be eligible for a trial specifically seeking this subtype. Of note, we did not double-count any HLA subtypes that were already part of a previously counted serotype. By summing the eligibility probabilities across all HLA subtypes, we arrived at the overall likelihood for African American individuals who qualify for any HLA-specific trial. In this case, it was largely driven by HLA-A2 serotype and totaled 33.0%.

### Statistical Analysis

Data were analyzed from May 8 to July 1, 2022. Analyses were descriptive. Calculations were performed using Microsoft Excel, 16.61 (22050700) (Microsoft Corporation).

## Results

### Cross-Sectional Analysis

Of 2135 trials, 263 met our inclusion criteria. The eFigure in Supplement 1 details the trial identification methods and selection process and reasons for exclusion.

Most trials studied an anticancer therapeutic strategy (258 [98.1%; 95% CI, 96.4%-99.7%]), whereas 4 (1.5%; 95% CI, 0.04%-3.0%) studied an antiviral strategy (3 against HIV and 1 against cytomegalovirus infection), and 1 trial (0.4%; 95% CI, −0.4%-1.1%) studied a treatment against neovascular maculopathy, including age-related macular degeneration. Most trials studied a therapeutic vaccine (179 [68.1%; 95% CI, 62.4%-73.7%]) or a cellular therapy (73 [27.8%; 95% CI, 22.3%-33.2%]).

Interventions were restricted to patients with 1 HLA-subtype in 217 trials (82.5%; 95% CI, 77.9%-87.1%) and more than 1 subtype in 46 (17.5%; 95% CI, 12.9%-22.1%). Other trial characteristics are described in Table 1. For trials studying an anticancer intervention (n = 258), we detailed which tumor type was under study, according to the mechanism of action of the intervention, in the eTable in Supplement 1.

**Table 1.** Characteristics of Trials Investigating a Therapeutic Intervention With Restricted Eligibility Based on HLA Subtype

Characteristic	No. (%) [95% CI] of trials (n = 263)
Mechanism of action	
Vaccine therapy	179 (68.1) [62.4 to 73.7]
Cellular therapy	73 (27.8) [22.3 to 33.2]
Fusion protein	8 (3.0) [1.0 to 5.1]
Other	3 (1.1) [−0.1 to 2.4]
Disease	
Cancer	258 (98.1) [96.4 to 99.7]
Infection	4 (1.5) [0.04 to 3.0]
Neovascular maculopathy	1 (0.4) [−0.4 to 1.1]
Type of restriction	
1 HLA subtype	217 (82.5) [77.9 to 87.1]
>1 HLA subtype	46 (17.5) [12.9 to 22.1]
HLA subtype^a^	
HLA-A*02:01	115 (43.7) [37.7 to 49.7]
HLA-A2	114 (43.3) [37.4 to 49.3]
HLA-A*24:02	31 (11.8) [7.9 to 15.7]
HLA-A3	13 (4.9) [2.3 to 7.6]
HLA-A*02:06	12 (4.6) [2.0 to 7.1]
HLA-A1	12 (4.6) [2.0 to 7.1]
HLA-A24	7 (2.7) [0.7 to 4.6]
HLA-A*02:05	6 (2.3) [0.5 to 4.1]
Trial status	
Completed	120 (45.6) [39.6 to 51.6]
Terminated	41 (15.6) [11.2 to 20.0]
Recruiting	39 (14.8) [10.5 to 19.1]
Unknown status	26 (9.9) [6.3 to 13.5]
Active, not recruiting	23 (8.7) [5.3 to 12.2]
Withdrawn	8 (3.0) [1.0 to 5.1]
Not yet recruiting	4 (1.5) [0.04 to 3.0]
Available	1 (0.4) [−0.4 to 3.0]
Suspended	1 (0.4) [−0.4 to 3.0]
Phase of trials	
1	91 (34.6) [28.9 to 40.3]
1-2	85 (32.3) [26.7 to 38.0]
2	72 (27.4) [22.0 to 32.8]
Early 1	6 (2.3) [0.5 to 4.1]
3	6 (2.3) [0.5 to 4.1]
2-3	2 (0.8) [−0.3 to 1.8]
Expanded access program	1 (0.4) [−0.4 to 3.0]

Abbreviation: HLA, human leukocyte antigen.

^a^ Only HLA subtypes found in at least 5 instances are represented.

All of the HLA subtypes found in at least 5 instances were A subtypes (HLA-A). The most identified HLA subtypes were the HLA-A*02:01 allele in 115 trials (43.7%; 95% CI, 37.7%-49.7%), the HLA-A2 serotype in 114 (43.3%; 95% CI, 37.4%-49.3%), the HLA-A*24:02 allele in 31 (11.8%; 95% CI, 7.9%-15.7%), and the HLA-A3 serotype in 13 (4.9%; 95% CI, 2.3%-7.6%) (Table 1).

### HLA Subtypes According to Ethnic Background

Because HLA subtypes found in at least 5 instances of included trials were exclusively in the HLA-A subgroup, we searched for the 4 most frequent HLA-A alleles in each of 7 racial and ethnic categories (CIWD, version 3.0.0, data). While the HLA-A*02:01 allele was the most frequent in 6 of 7 categories, its frequency varied from 11.9% (95% CI, 11.8%-12.0%) in individuals of African or African American descent to 27.1% (95% CI, 27.1%-27.1%) in individuals of European or European descent (Table 2). Among the Asian or Pacific Islander populations, this subtype was not among the top 4 most frequent subtypes and was present in only 6.5% (95% CI, 6.5%-6.5%) of individuals’ alleles.

**Table 2.** Most Frequent HLA-A Alleles in the Overall Population by Race and Ethnicity

HLA-A subtype^a^	Allele frequency, % (95% CI)							
	Total	African or African American	Asian or Pacific Islander	European or European descent	Middle Eastern or NorthernAfrican	South or Central American, Hispanic, or Latino	American Indian or Alaska Native	Unknown, not asked, multiple ancestries, or other
A*02:01	24.1 (24.0-24.1)	11.9 (11.8-12.0)	6.5 (6.5-6.5)	27.1 (27.1-27.1)	17.4 (17.2-17.5)	19.9 (19.8-20.0)	19.4 (19.1-19.7)	21.8 (21.7-21.9)
A*01:01	13.3 (13.3-13.4)	4.2 (4.1-4.3)	10.6 (10.6-10.7)	14.6 (14.6-14.6)	10.5 (10.4-10.6)	6.9 (6.8-6.9)	7.2 (7.0-7.4)	12.2 (12.2-12.3)
A*03:01	11.9 (11.8-11.9)	7.6 (7.5-7.6)	4.9 (4.9-5.0)	13.3 (13.3-13.4)	8.0 (7.9-8.1)	7.1 (7.0-7.1)	7.3 (7.1-7.5)	10.4 (10.3-10.4)
A*24:02	8.9 (8.9-9.0)	2.4 (2.4-2.5)	14.4 (14.4-14.5)	8.4 (8.4-8.4)	12.1 (12.0-12.2)	10.8 (10.8-10.9)	10.3 (10.1-10.5)	8.7 (8.6-8.7)

Abbreviation: HLA, human leukocyte antigen.

^a^ Based on the catalog of Common, Intermediate, and Well-Documented HLA Alleles in World Populations, version 3.0.0.^4^

### Likelihood of Being Enrolled in Trials

The likelihood of being enrolled in any of the selected trials varied according to racial and ethnic group, vis-à-vis differences in HLA subtype estimated prevalence. Being of African or African American descent conferred the lowest chance (33.0%) of enrollment compared with being of European descent (53.0%). Detailed results for each race category are reported in Table 3.

**Table 3.** Likelihood (Highest Estimation) and Relative Likelihood of Eligibility in Trials Investigating a Therapeutic Intervention With Restricted Eligibility Based on Human Leukocyte Antigen Subtype^a^

Eligibility	Race or ethnicity							
	African or African American	Asian or Pacific Islander	European or European descent	Middle Eastern or North African	South or Central American, Hispanic, or Latino	American Indian or Alaska Native	Unknown, not asked, multiple ancestries, or other	All populations
Likelihood (highest estimation), %	33.0	36.3	53.0	41.6	50.4	49.2	47.3	50.3
Relative likelihood as compared with all populations	0.66	0.72	1.05	0.83	1.00	0.98	0.94	1 [Reference]

^a^ The likelihood of eligibility is the combination of 2 proportions; hence, the 95% CIs were not calculated.

**Figure 1.**
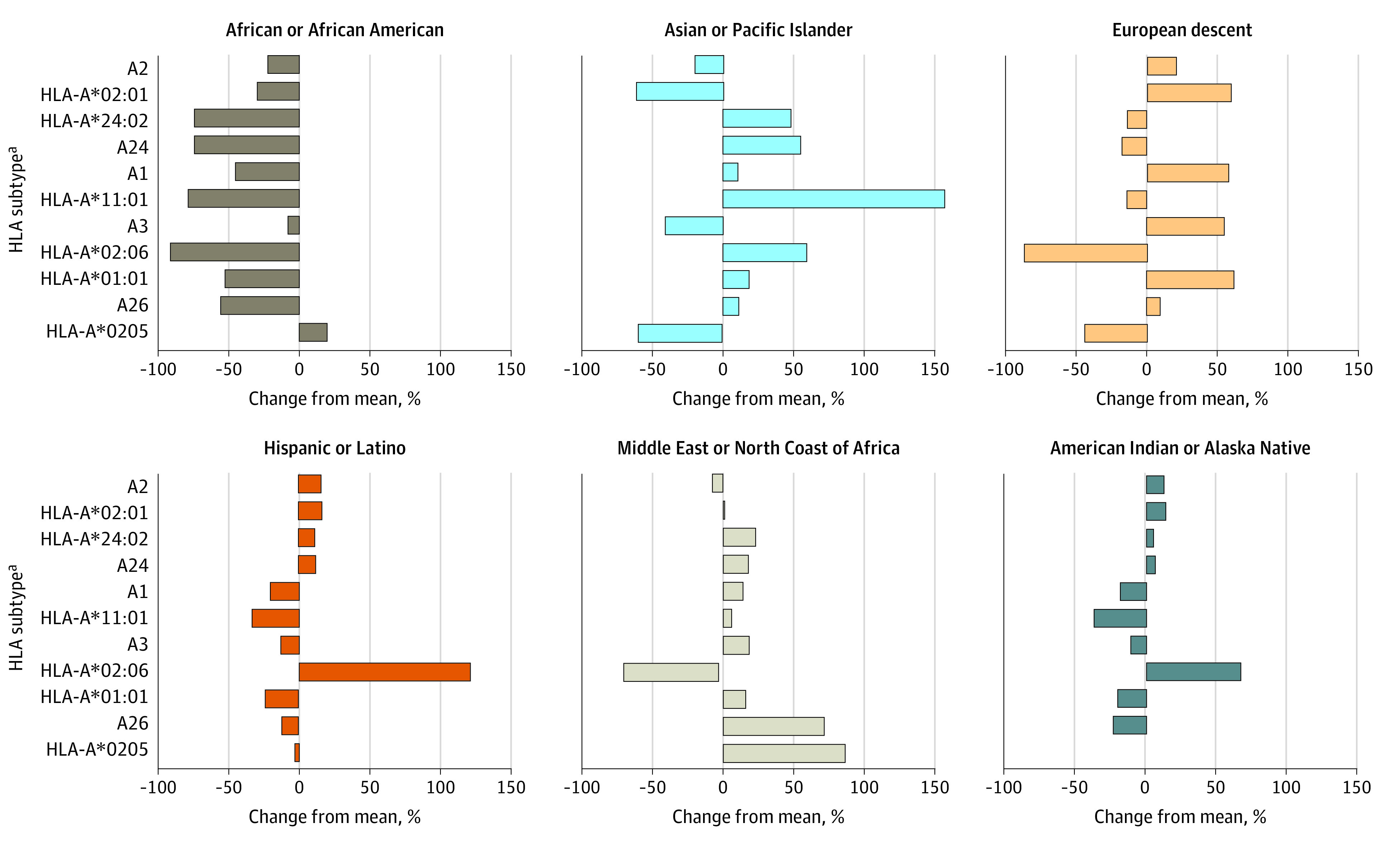
Percentage Difference of Human Leukocyte Antigen (HLA) Subtype Frequencies Between Each Racial and Ethnic Group and the Frequencies Among the Overall Population ^a^Includes HLA subtypes identified in more than 5 trials.

For HLA subtypes represented in more than 5 trials, the percentage deviation of the racial and ethnic groups’ subtype frequency from the overall population mean of the subtype frequency is represented in Figure 1. A percentage change greater than 0 indicates a greater representation of the subtype among the racial or ethnic group than the overall population.

**Figure 2.**
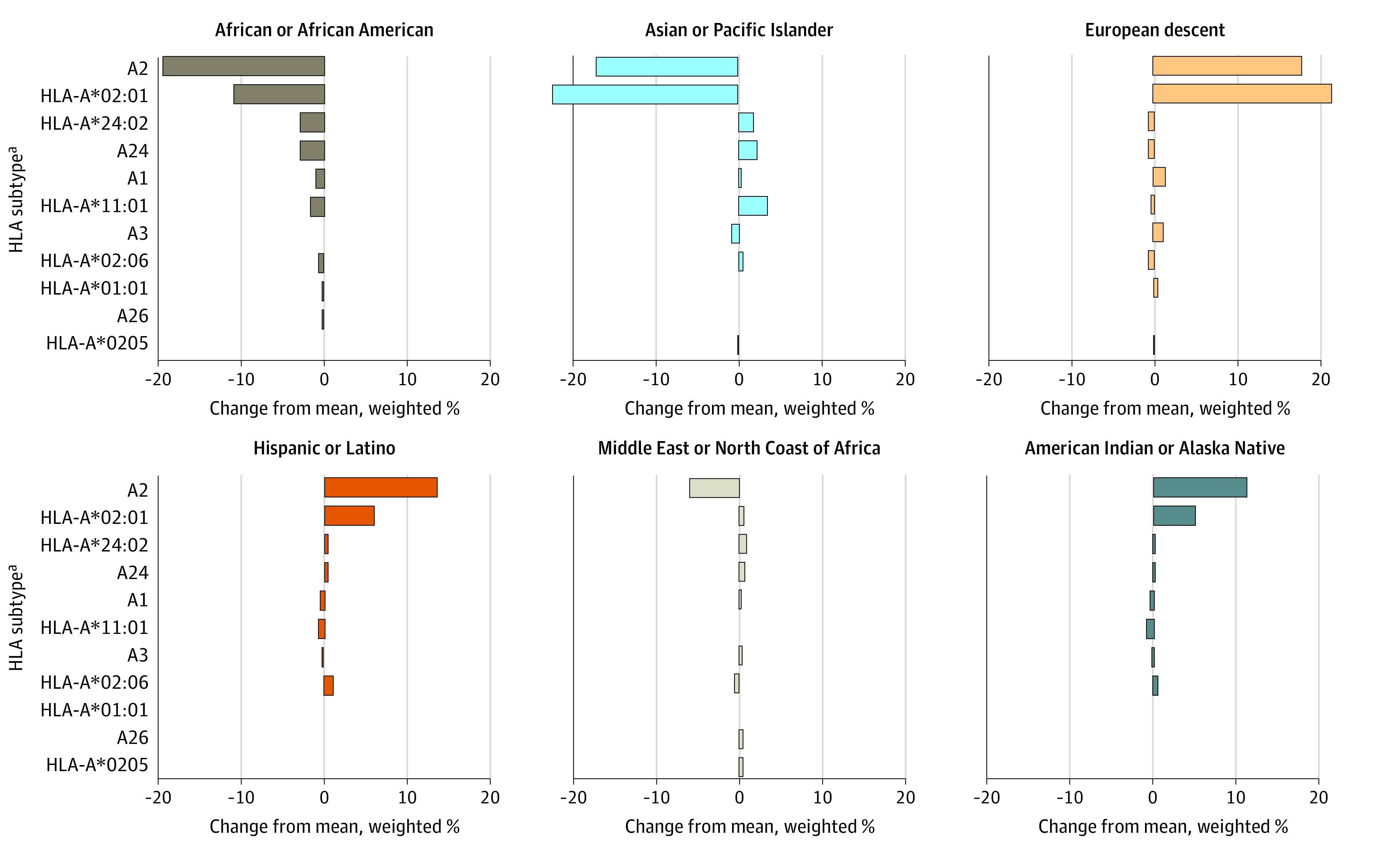
Percentage Difference of Human Leukocyte Antigen (HLA) Subtype Frequencies Between Each Racial and Ethnic Group and the Frequencies Among the Overall Population, Weighted by Percentage of Slots Available in Trials ^a^Includes HLA subtypes identified in more than 5 trials.

In Figure 2, we illustrated the percentage deviation of each subtype frequency by race or ethnicity from the overall population, weighted by percentage of slots available in trials. An unequal access to trials according to racial or ethnic background is visible by scores that are skewed toward negative values for African or African American and Asian or Pacific Islander groups compared with values skewed toward positive values in populations of European descent and American Indian or Alaska Native, and South or Central American, Hispanic, or Latino populations.

### Racial and Ethnic Representation in Selected Trials

From our selected trials, we identified those that were completed and had results displayed on ClinicalTrials.gov, which reports racial categories. All 27 trials had either the HLA-A2 serotype or the HLA-A*02:01 subtype as an inclusion criterion. We further excluded trials where racial and ethnic categories were not explicitly reported. Among 11 completed trials describing racial categories of patients, 326 patients were enrolled; of these, 5 (1.5%) were categorized as Asian, 18 (5.5%) as Black, 301 (92.3%) as White, and 2 (0.6%) as unknown.

## Discussion

We found 263 trials that studied a therapeutic strategy in participants with specific HLA subtypes. Because the prevalence of HLA subtypes varies according to an individual’s racial or ethnic background, we estimated that individuals of African American descent had a 33.0% chance of being eligible for 1 such trial, while those of European descent had a 53.0% chance. The 60% higher likelihood among those of European descent, compared with those of African American descent, may be the first evidence of structural bias in the drug development programs of HLA-restricted therapies.

The recent approval of tebentafusp among patients with advanced or metastatic uveal melanoma may be illustrative.^5^ Tebentafusp was the first approved compound targeting patients bearing a specific HLA subtype and demonstrated an overall survival benefit against the treatment of physician’s choice in the IMCgp100-202 trial.^6^ To be eligible for the treatment, patients needed to have the HLA-A02:01 allele, and only 51.4% of screened patients met eligibility. In a US cohort, the HLA-A2 subtype was present in 35% of African American individuals and 50% of White individuals.^15^ In participants with HLA-A2, the HLA-A*02:01 subtype was the most common, with variation across racial groups (53% in Asian or Pacific Islander individuals and ≤96% in White individuals).^15^ Therefore, from an individual patient perspective, being diagnosed with uveal melanoma offers unequal opportunities to HLA-restricted therapies according to one’s racial and ethnic background.

On the other hand, it has been shown in the US that the incidence of uveal melanoma is higher among White individuals.^16^ Factors explaining this difference are mostly unknown, but hypotheses about different UV light exposure or genetic predisposition have been proposed.^17^ If an HLA restriction must be used by a drug developer, there may be a rationale to target the population that is most likely to present the disease under investigation. Financial incentives may also drive companies to target the largest market share. However, because of an underrepresentation of minority populations, this may lead to discordance between the restriction and the principle of equal access to innovation. This may be even more relevant when considering this issue globally, and across all drugs.

Consider also the case of prostate cancer. African American individuals present with a higher incidence of localized or metastatic disease than White individuals.^18^ Our work identified 11 trials focusing specifically on this tumor type,^19,20,21,22,23,24,25,26,27,28,29^ 10 of which (90.9%) targeted populations with HLA-A2 serotype or HLA-A*02:01 allele, which is less prevalent in individuals of African descent than in those of European decent. In this case, the drug development pipeline is offering less opportunity for treatment to the more affected population.

Recruiting underrepresented minority populations to participate in clinical trials has long been a challenge, as these groups often face barriers ranging from mistrust of medical institutions to socioeconomic constraints.^30,31^ Our results suggest that strict subtyping or genetic requirements in clinical trials can exacerbate these challenges. When studies mandate specific biomarkers, it can unintentionally exclude those from diverse ethnic and genetic backgrounds who might not exhibit the exact criteria but still could benefit from or contribute to the research. This not only diminishes the diversity of the participant pool, it also reduces the generalizability of the trial’s findings. In a subset of trials of HLA-based therapeutics, we found that only 25 of 326 enrolled patients (7.7%) were identified as being a race other than White. While the low representation may be multifactorial, the HLA restriction is a possible contributing factor.

In medicine, some HLA subtypes are associated with specific disease susceptibility^32^ or drug-induced adverse reactions.^33,34^ In heme tumors, HLA matching between donors and receivers is critical when considering allogeneic stem cell transplantation strategies,^35^ as well as in solid organ transplantation.^36^ In therapeutic drug development, HLA restriction emerged as a technical and biological constraint.^37^ Epitope-based therapeutic vaccines may rely on epitopes expressed by specific HLA subtypes.^38^ The choice of the epitopes that the vaccine will target naturally leads to a restriction of the therapy to a population with the specific HLA subtype. In other cases, the therapeutic product was developed from a patient, and the HLA restriction followed that of the patient. As an example, 1 trial included in our analysis investigated DMF5 cells.^39^ Those cells, initially collected from a patient with the HLA-A:02:01 subtype, are T cells genetically engineered to express an HLA-A*0201-restricted T-cell receptor used within a clinical trial of adoptive transfer therapy.^40^

With a significant number of therapeutics being developed to target populations with specific HLA subtypes, issues of restriction should be addressed and, if possible, overcome. Overcoming HLA restriction may be achievable by various techniques but is still a challenging task and is not always possible.^37^ During peptide identification processes, researchers may eventually be aware of racial disparities posed by the restriction. In their work, Stanojevic et al^38^ described peptide identification with HLA subtypes that are common in African American, Asian, Hispanic, and Pacific Islander populations. They concluded that “defining PRAME [Preferentially Expressed Antigen in Melanoma]-specific T cells beyond HLA-A*02-restricted epitopes could be useful when developing T-cell therapeutics for worldwide application”.^38^ For researchers, being aware that HLA restriction can introduce racial inequality in HLA-based therapeutics could motivate them to systematically incorporate and evaluate techniques to overcome such restrictions, whenever possible. There is a need to prevent or lessen unequal access to innovation worldwide based on racial, ethnic, and geographical origins.

### Strengths and Limitations

This study has several strengths. First, to our knowledge, it is the first to examine whether drug development programs based on HLA restriction may constitute a structural bias in biomedicine. We encourage other investigators to examine this question as well. Second, the data were reviewed independently by 2 reviewers. Third, we provide racial and ethnic perspectives of our findings. Last, we used a comprehensive database for our work (CIWD, version 3.0.0).

In terms of limitations, our analysis was conducted on trials from ClinicalTrials.gov, and some trials may not have been registered on this platform. Additionally, the impact of HLA-restriction in current therapeutic strategies in medicine remains limited. Indeed, only 1 compound is approved in a relatively rare tumor type (uveal melanoma). Finally, we did not analyze the racial and ethnic breakdown of every disease state for which drugs are being developed. This is both because such a project would be overly time consuming, but also because for some specific, niche indications, there are no reliable data on the racial and ethnic breakdown, especially when specific inclusion criteria were applied. We encourage others to develop this work and investigate this pressing issue.

## Conclusions

This cross-sectional study identified 263 clinical trials testing a therapeutic strategy that targets patients with 1 or more HLA subtype. Most trials investigated an anticancer therapy, and most were vaccine therapeutics. Most trials restricted the intervention to patients with the HLA-A2 or the HLA-A02:01 (a subset of HLA-A2) subtypes. Both subtypes are more prevalent in individuals who are of American Indian or Alaska Native, European, or Hispanic descent than in individuals of other racial and ethnic groups, such as African American or Asian populations. We find systemic racial and ethnic inequity posed by HLA-restricted strategies. Specifically, an individual of African American descent has a 33.0% likelihood of participation in a trial, while an individual of European descent has a 53.0% likelihood. Overcoming HLA restrictions poses biological challenges, but solutions must be introduced and implemented, especially considering the task of the research community to provide equal access to innovative strategies regardless of race or ethnicity.

## Notes

Supplement 1. **eTable.** Tumor Types, According to the Mechanism of Action Under Investigation, in Selected Trials Studying an Anticancer Therapy (n = 258) **eFigure.** Flowchart of the Trial Selection Process and Reasons for Exclusion

Supplement 2.
Data Sharing Statement
