# Supplementary material for: Eligibility for Human Leukocyte Antigen–Based Therapeutics by Race and Ethnicity
Source: JAMA Netw Open. 2023 Oct 26;6(10):e2338612. doi: 10.1001/jamanetworkopen.2023.38612 (PMC10603498; doi:10.1001/jamanetworkopen.2023.38612)
Supplement: Supplement 1. — eTable. Tumor Types, According to the Mechanism of Action Under Investigation, in Selected Trials Studying an Anticancer Therapy (n = 258) eFigure. Flowchart of the Trial Selection Process and Reasons for Exclusion [file jamanetwopen-e2338612-s001.pdf]

Supplementary Online Content

Olivier T, Haslam A, Tuia J, Prasad V. Eligibility by Race and Ethnicity for Human Leukocyte Antigen–Based therapeutics. *JAMA Netw Open*. 2023;6(10):e2338612. doi:10.1001/jamanetworkopen.2023.38612

eTable. Tumor Types, According to the Mechanism of Action Under Investigation, in Selected Trials Studying an Anticancer Therapy (n=258)
eFigure. Flowchart of the Trial Selection Process and Reasons for Exclusion

This supplementary material has been provided by the authors to give readers additional information about their work.

© 2023 Olivier T, et al. *JAMA Network Open*.

eTable. Tumor Types, According to the Mechanism of Action Under Investigation, in Selected Trials Studying an Anticancer Therapy (n=258)

	**Cellular Therapy**	**Fusion Protein**	**Other**	**Vaccine Therapy**	**Total**
	**(N=71)**	**(N=8)**	**(N=3)**	**(N=176)**	**(N=258)**
**Tumor type**					
Central Nervous System	1 (1.4%)	0 (0%)	0 (0%)	16 (9.1%)	17 (6.6%)
Cervical Cancer	1 (1.4%)	0 (0%)	0 (0%)	0 (0%)	1 (0.4%)
Esophageal Cancer	2 (2.8%)	0 (0%)	0 (0%)	4 (2.3%)	6 (2.3%)
Head and Neck	3 (4.2%)	1 (12.5%)	0 (0%)	1 (0.6%)	5 (1.9%)
Heme Malignancies	9 (12.7%)	0 (0%)	1 (33.3%)	12 (6.8%)	22 (8.5%)
Kidney Cancer	1 (1.4%)	0 (0%)	0 (0%)	2 (1.1%)	3 (1.2%)
Liver Cancer	4 (5.6%)	0 (0%)	0 (0%)	2 (1.1%)	6 (2.3%)
Many Tumor Types	25 (35.2%)	4 (50.0%)	0 (0%)	14 (8.0%)	43 (16.7%)
Melanoma	10 (14.1%)	3 (37.5%)	0 (0%)	54 (30.7%)	67 (26.0%)
Non-Small Cell Lung Cancer	2 (2.8%)	0 (0%)	2 (66.7%)	13 (7.4%)	17 (6.6%)
Other	3 (4.2%)	0 (0%)	0 (0%)	1 (0.6%)	4 (1.6%)
Ovarian Cancer	4 (5.6%)	0 (0%)	0 (0%)	5 (2.8%)	9 (3.5%)
Pancreatic Cancer	1 (1.4%)	0 (0%)	0 (0%)	6 (3.4%)	7 (2.7%)
Sarcoma	5 (7.0%)	0 (0%)	0 (0%)	0 (0%)	5 (1.9%)
Bladder Cancer	0 (0%)	0 (0%)	0 (0%)	1 (0.6%)	1 (0.4%)
Breast Cancer	0 (0%)	0 (0%)	0 (0%)	23 (13.1%)	23 (8.9%)
Colorectal Carcinoma	0 (0%)	0 (0%)	0 (0%)	5 (2.8%)	5 (1.9%)
Gastric Cancer	0 (0%)	0 (0%)	0 (0%)	4 (2.3%)	4 (1.6%)
Prostate Cancer	0 (0%)	0 (0%)	0 (0%)	11 (6.3%)	11 (4.3%)
Renal Cell Carcinoma	0 (0%)	0 (0%)	0 (0%)	1 (0.6%)	1 (0.4%)
Small Cell Lung Cancer	0 (0%)	0 (0%)	0 (0%)	1 (0.6%)	1 (0.4%)

© 2023 Olivier T, et al. *JAMA Network Open*.

eFigure. Flowchart of the Trial Selection Process and Reasons for Exclusion

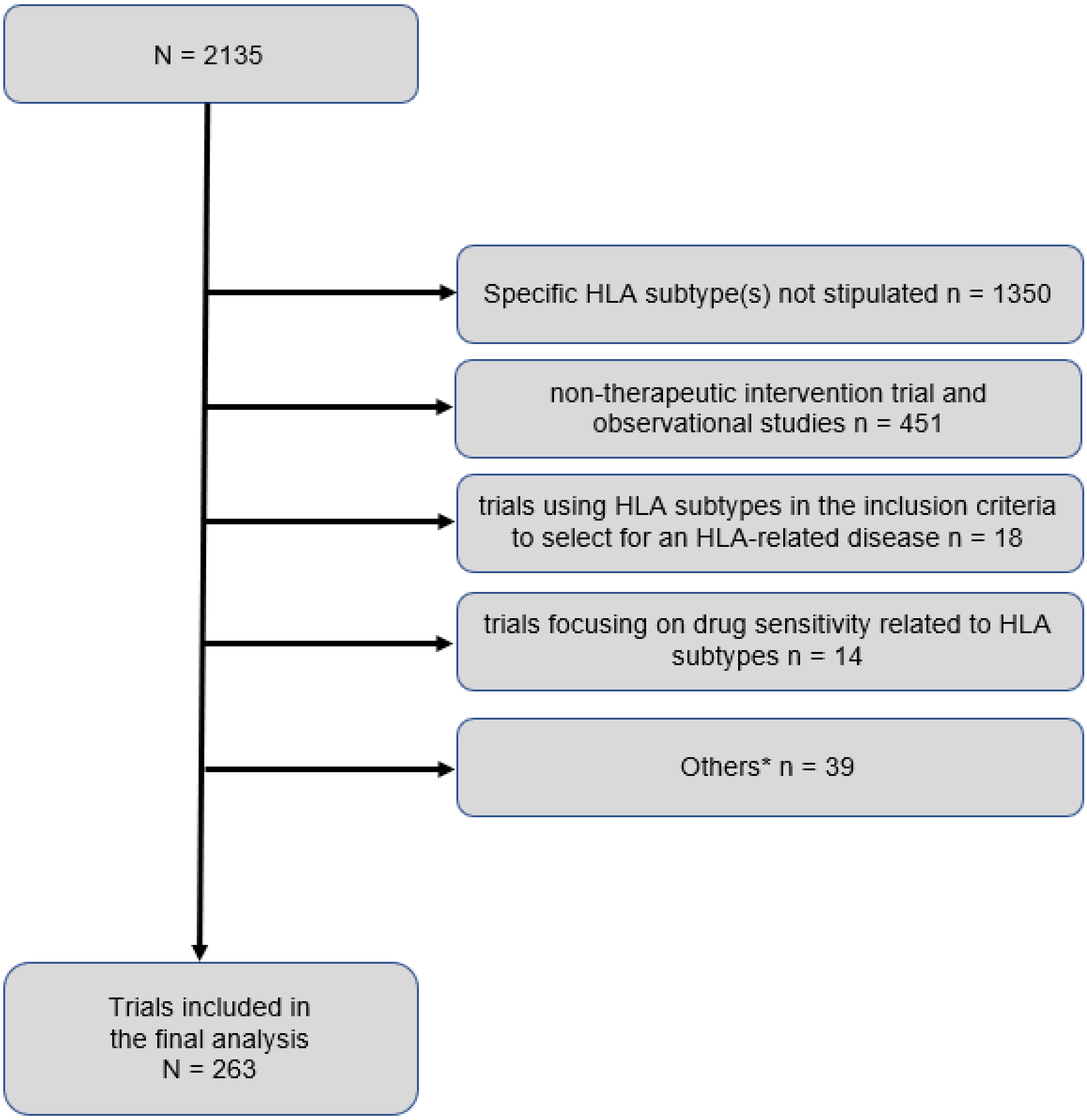

* Among those: 34 trials studied HLA as a biomarker, 2 referred to HLA as hyaluronic acid, and 3 others had no relation with classical HLA subtypes.

© 2023 Olivier T, et al. *JAMA Network Open*.
