# Supplementary material for: Eligibility for Human Leukocyte Antigen–Based Therapeutics by Race and Ethnicity
Source: JAMA Netw Open. 2023 Oct 26;6(10):e2338612. doi: 10.1001/jamanetworkopen.2023.38612 (PMC10603498; doi:10.1001/jamanetworkopen.2023.38612)
Supplement: Supplement 2. — Data Sharing Statement [file jamanetwopen-e2338612-s002.pdf]

Data Sharing Statement

Olivier. Eligibility for Human Leukocyte Antigen–Based Therapeutics by Race and Ethnicity. *JAMA Netw Open*. Published October 26, 2023. doi:10.1001/jamanetworkopen.2023.38612

Data
Data available: Yes
Data types: Other (please specify)
Additional Information: All data on which this work was based are publicly available. The data generated during the study are available upon reasonable request from the corresponding author.
How to access data: From the corresponding author (timothee.olivier@hcuge.ch)
When available: With publication

Supporting Documents
Document types: Other (please specify)
Additional Information: Spreadsheet of the data generated.
How to access documents: From the corresponding author (timothee.olivier@hcuge.ch)
When available: With publication

Additional Information
Who can access the data: Anyone requesting the data.
Types of analyses: For any purpose.
Mechanisms of data availability: From timothee.olivier@hcuge.ch, spreadsheet of the data generated.
